# A Case Report of a Novel Myelin Protein Zero (*MPZ*) Pathogenic Variant in Charcot–Marie–Tooth Disease Combined With Type 2 Diabetes

**DOI:** 10.1155/crig/8838487

**Published:** 2026-09-12

**Authors:** Lu Xia, Liyan Cai, Xin Chen, Kun Zhou, Wei Huang, Yuanyuan Zhu, Shinian Yang

**Affiliations:** ^1^ Department of Rehabilitation, Chengdu First People’s Hospital, Chengdu Sichuan, 610041, China, scu.edu.cn; ^2^ Department of Rehabilitation, Chengdu Integrated TCM&Western Medicine Hospital, Chengdu Sichuan, 610041, China, scu.edu.cn; ^3^ Department of Neurology, Chengdu First People’s Hospital, Chengdu Sichuan, 610041, China, scu.edu.cn; ^4^ Department of Neurology, Chengdu Integrated TCM&Western Medicine Hospital, Chengdu Sichuan, 610041, China, scu.edu.cn

**Keywords:** Charcot–Marie–tooth disease, *MPZ* gene mutation, myelin protein zero, type 2 diabetes

## Abstract

Charcot–Marie–Tooth (CMT) disease is the collective term for the most common inherited peripheral neuropathies, affecting both motor and sensory nerves. A typical CMT patient presents with slowly progressive distal muscle weakness and atrophy that primarily involves the small foot muscles, peroneal muscles, and, often later, the muscles of the hands and forearms. Foot deformities, most commonly pes cavus and claw toes, are common and can lead to gait impairments. In this paper, we report a patient with an intermediate CMT (CMT‐Int) type carrying a novel pathogenic variant in the *MPZ* gene combined with type 2 diabetes. The patient’s initial symptoms included a gradual onset of muscle wasting, weakness, and sensory impairment in the lower limbs. Electrophysiological findings suggested widespread peripheral nerve damage affecting both sensory and motor fibers, with a predominant demyelinating pattern accompanied by axonal damage. Genetic testing identified a previously unreported heterozygous mutation in the *MPZ* gene, specifically c.548G > A, p.Trp183∗. This alteration results in the replacement of the 183rd amino acid (tryptophan) by a stop codon. This premature stop codon is predicted to escape nonsense‐mediated mRNA decay, resulting in a C‐terminally truncated protein that may exert a dominant‐negative effect. Although a different variant affecting the same codon (c.549G > A, p.Trp183∗, VCV000917145.1) has been documented, this particular pathogenic mutation has not been previously recorded. After being diagnosed with CMT, the patient developed type 2 diabetes following pancreatic cyst surgery in September 2022. The presence of both conditions exposed her peripheral nerves to congenital structural abnormalities combined with the metabolic consequences of chronic hyperglycemia and local ischemia, thereby exacerbating nerve injury.

## 1. Introduction

Charcot–Marie–Tooth (CMT) disease is a major neurological disorder with a prevalence of approximately 1 in 2500 and shows significant racial disparities in its occurrence [1]. Since its initial description by Charcot, Marie, and Tooth in 1886, the core clinical features of this syndrome have been defined as distal peroneal weakness accompanied by muscular atrophy [2]. Today, the classification of CMT syndromes has been revised and extended based on clinical, electrophysiological, histopathological, and genetic findings [3], guided first by advances in clinical electrophysiology and later by molecular genetics. Over 90 genes have been implicated in CMT (https://www.musclegenetable.fr/index.html), reflecting its marked genetic heterogeneity. These mutations affect proteins involved in the structural integrity and function of the myelin sheath or axons in peripheral nerves, leading to myelin or axonal dysfunction. They may also cause additional manifestations, including sensorineural hearing loss, vocal cord paresis, deafness, and intellectual disability. The disease can be inherited in an autosomal dominant, autosomal recessive, or X‐linked recessive manner. The relative frequency of CMT subtypes varies substantially by ancestry and geographic region [4].

CMT is further classified into subtypes based on nerve conduction velocity (NCV), inheritance pattern, and causative genes. The primary classification divides CMT into demyelinating CMT disease type 1 (CMT1, NCV < 38 m/s), axonal CMT disease type 2 (CMT2, NCV > 38 m/s), and intermediate CMT (CMT‐Int, NCV 25–45 m/s). The most common subtype in European populations is CMT disease type 1A (CMT1A), caused by a duplication of the *PMP22* gene, accounting for approximately 40%–50% of all CMT cases. Other notable subtypes include CMT disease type 1B (CMT1B), caused by *MPZ* mutations; CMT disease type 2A (CMT2A), caused by *MFN2* mutations, the most common axonal form; and CMT disease type X1 (CMTX1), caused by *GJB1* mutations, the latter being particularly frequent in Japan. CMT disease type 4 (CMT4) subtypes are rare and often more severe than CMT1 or CMT2. Distal hereditary motor neuropathy (dHMN) is a related disorder that primarily affects motor nerves. Historical designations, such as CMT disease type 3 (CMT3), also called Dejerine–Sottas syndrome (DSS) and CMT5‐7, are no longer used as primary classifications. Accurate subtyping is essential for prognosis, genetic counseling, and guiding targeted molecular diagnosis [5, 6]. Based on inheritance, CMT1 and CMT2 are typically autosomal dominant, CMT4 is autosomal recessive, and CMTX is X‐linked.

In this case report, we describe a patient with CMT‐Int carrying a novel pathogenic mutation in the *MPZ* gene (NM_000530.6 c548G > A) on 1q23, exon 4. This patient also had type 2 diabetes mellitus (DM). The coexistence of both diseases subjected her peripheral nerves to both congenital structural deficits and the combined metabolic insults of chronic hyperglycemia and local ischemia, leading to aggravated nerve injury.

## 2. Clinical Case Description

### 2.1. First Admission (November 2018)

A 43‐year‐old female patient was admitted to the Neurology Department of Huaxi Hospital in November 2018 due to bilateral foot dorsiflexion weakness and an unstable gait that had persisted for more than two years. Her symptoms began with left foot dorsiflexion weakness, which was later accompanied by heel weakness, gait instability, mild atrophy of the left lower leg, and deep sensory impairment in the lower limbs. She had no deafness or other neurological symptoms and no family history of similar neurological conditions. On admission, her vital signs were stable. She was conscious and alert, without signs of distress or jaundice. Cardiopulmonary and abdominal examinations revealed no significant abnormalities.

### 2.2. Neurological Examination

Neurologically, she was alert with clear speech and normal higher cortical functions. Cranial nerve examination showed strong bilateral eye closure; pupils measured 2.5 mm on the left and 3.0 mm on the right, with brisk direct and consensual light reflexes. Extraocular movements were full, facial features were symmetrical, tongue protrusion was in the midline without tremor or atrophy, and the neck was supple. Gait examination revealed left foot drop and unstable heel walking. Mild muscle atrophy was noted in the left lower leg, while muscle tone was normal in all four limbs. Muscle strength was 5/5 in the left upper limb and in the proximal right upper limb, with mild reduction in finger‐to‐finger pinch strength. In the lower limbs, strength was as follows: left iliopsoas and quadriceps 4+/5, left hamstrings 4/5, right iliopsoas 4+/5, left foot dorsiflexion 1/5, and right foot dorsiflexion 3/5. Coordination was adequate on finger‐to‐nose and heel‐knee‐shin tests; the Romberg test was negative, but tandem gait was performed poorly. Sensory examination demonstrated symmetrical, normal pain sensation and intact vibration and position sense. Deep tendon reflexes were normal in the upper limbs, diminished at both knees, and absent at both ankles. Jaw jerk was equivocal, while the palmar reflex and Babinski sign were negative bilaterally. No meningeal signs were observed.

### 2.3. Examination

After admission, relevant examinations were performed. Complete blood count, erythrocyte sedimentation rate, coagulation profile, liver and kidney function tests, lipid profile, blood glucose, glycosylated hemoglobin, homocysteine, creatine kinase, thyroid function and antibodies, folate, vitamin B12, amylase, and lipase were all within normal limits. Electrocardiography and echocardiography revealed no significant abnormalities.

Lumbar puncture examination revealed an opening pressure of 140 mmH_2_O and a closing pressure of 45 mmH_2_O. Routine cerebrospinal fluid (CSF) analysis, biochemistry, and IgG synthesis rate were all normal, and etiological smears were negative. MRI of the brain, cervical, thoracic, and lumbar spines showed normal signal intensity of the brain and spinal cord, with evidence of cervical and lumbar intervertebral disk herniation.

Electromyography (EMG) examination revealed significant abnormalities. The sensory nerve action potential (SNAP) amplitude of the left sural nerve was reduced. No SNAP was elicited from the right sural, right ulnar, or right median nerve. The motor conduction compound muscle action potential (CMAP) of the bilateral common peroneal nerves was not detected. The distal motor latency (DML) of the motor conduction CMAP for the bilateral tibial nerves was significantly prolonged, with a marked decrease in motor nerve conduction velocity (MNCV) and a significant prolongation of F‐wave latencies in the detected upper and lower limb nerves. The F‐wave emergence rate of the right median nerve was decreased, and the H reflex of the bilateral tibial nerves was not elicited. Neurogenic damage was observed in the right anterior tibial muscle and the inner head of the left gastrocnemius muscle, while the right biceps muscle showed no significant abnormalities. These electrophysiological findings suggest widespread peripheral nerve damage affecting both sensory and motor fibers, with a predominant demyelinating pattern accompanied by axonal damage.

DNA was extracted from peripheral blood using a column‐based method. A panel of 29 genes associated with inherited peripheral neuropathies (*AARS*, *AIFM1*, *BSCL2*, *DYNC1H1*, *EGR2*, *FGD4*, *FIG4*, *GARS*, *GDAP1*, *GJB1*, *HSPB1*, *HSPB8*, *KIF1B*, *LITAF*, *LMNA*, *LRSAM1*, *MED25*, *MFN2*, *MPZ*, *MTMR2*, *NDRG1*, *NEFL*, *PMP22*, *PRPS1*, *PRX*, *RAB7A*, *SBF2*, *SH3TC2*, and *TRPV4*) was sequenced. The obtained sequences were compared with reference sequences to identify potential pathogenic variants. Multiplex ligation‐dependent probe amplification (MLPA) was performed to analyze each exon of the *PMP22* gene, using healthy control DNA as a reference, to detect deletions or duplications. The identified *MPZ* variant was annotated using the GRCh37/hg19 reference genome and the NM_000530.6 transcript. Genetic testing confirmed a pathogenic mutation in the *MPZ* gene (c.548G > A), consistent with the diagnosis of CMT disease.

### 2.4. Initial Treatment

The patient was treated with mecobalamin and vitamin B1, followed by acupuncture, moxibustion, and other symptomatic treatments at a local clinic. She reported no improvement in her symptoms, and her condition worsened, with increasing weakness in foot dorsiflexion. In light of these findings, the patient was admitted to our department in July 2024 for further treatment to manage her symptoms, slow disease progression, and improve her quality of life.

The patient has a history of pure hypercholesterolemia. In September 2022, she developed hyperglycemia following pancreatic cyst surgery. After the surgery, she was diagnosed with type 2 diabetes and has since taken dapagliflozin 10 mg once daily, alogliptin benzoate 25 mg once daily, and epalrestat 50 mg three times daily.

On readmission, neurological examination showed clear consciousness, cooperative behavior, accurate answers to questions, and clear speech. Higher cortical functions, including logic, judgment, comprehension, attention, memory, and calculation, were normal. Pupils were symmetrical and equally large, with a diameter of approximately 3 mm, and reactive to light. Forehead and nasolabial folds were symmetrical, tongue extension was midline without tremors or atrophy, and the neck was soft. Nystagmus was negative. The bilateral finger‐to‐nose test was stable; bilateral pes cavus was noted. During walking, the left foot was in a full foot‐drop position, and heel walking was unstable. Mild atrophy of the left lower leg was observed. Proximal muscle strength was grade 5 in both lower limbs. Dorsiflexion strength was grade 1 in both feet. Plantarflexion strength was grade 5 in both ankles. Muscle strength was grade 5 in both upper limbs. Pes cavus with calf stork‐leg‐like atrophy suggests the presence of characteristic clinical manifestations of CMT disease. The passive range of motion of the ankle joint was not limited. Limb muscle tone was normal. Bilateral pain sensation was symmetrical and normal. Vibration and position sense were intact bilaterally. Limb tendon reflexes were reduced. Bilateral finger‐to‐nose and heel‐knee‐shin tests were negative. The Romberg sign was negative. The tandem gait test could not be performed adequately. The left Babinski sign was positive.

### 2.5. Readmission Examination

After readmission, relevant examinations were completed. Biochemical tests showed glucose 6.13 mmol/L and glycosylated hemoglobin 6.4%. Complete blood count, electrolytes, blood lipids, coagulation studies, syphilis, HIV, and hepatitis B markers were within normal limits. A plain chest CT showed small nodules in both lungs, possibly representing chronic inflammation.

EMG revealed abnormal spontaneous potentials in the left gastrocnemius muscle, right anterior tibialis muscle, and abductor pollicis brevis muscle, with a general decrease in motor unit potential (MUP) number and an increase in MUP wave width in each muscle. Notable changes were observed in the peripheral nerves of both upper and lower limbs. Motor and sensory conduction velocities (MCV and SCV) were reduced (range: 23.1–40 m/s), indicating a demyelinating process affecting the nerves. The CMAP and SNAP amplitudes were decreased, indicating axonal damage. F‐wave latencies were prolonged (Table 1).

**TABLE 1:** Electromyography results of the patient (the second admission).

**Multi-MUP**									
**Muscle**		**Sweeps**	**Ampl. μV**	**Dur.ms**	**Phases**	**Turns**	**Area μVms**	**Ratio**	**Rate Hz**

Right tibialis anterior muscle	Mean ALL		1272.9	9.31	4.2	5	1666.7	1.41	12
	Total MUPs	25							
	%Polyphasic	20%							
Left gastrocnemius	Mean ALL		702.4	6.76	3.5	3.7	827.6	1.25	8
	Total MUPs	26							
	%Polyphasic	8%							
Right abductor pollicis brevis	Mean ALL		783.8	7.15	3.7	4	872.6	1.19	9
	Total MUPs	32							
	%Polyphasic	16%							

**Sensory nerve conduction velocity, SCV**									
**Nerve/sites**	**Rec. site**	**Onset lat ms**	**Peak lat ms**		**Amp μV**		**Distance mm**	**Velocity m/s**	

Right median nerve	Dig II	3.75	5.10		21.6		145	39	
Right ulnar nerve	Dig V	4.22	4.22		10.8		130	31	
Right sural nerve	Ankle	3.44	4.64		3.9		122	35	
Left sural nerve	Ankle	4.06	5.42		4.5		125	31	
Right superficial peroneal nerve	Ankle	3.75	4.53		1.7		107	29	
Left superficial peroneal nerve	Ankle	4.01	4.95		3.4		100	25	

**Compound muscle action potential, CAMP**									
**Nerve/sites**	**Muscle**	**Latency ms**	**Amp mV**	**Distance mm**		**Lat diff ms**		**Velocity m/s**	

Right median nerve									
Wrist	APB	5.31	7.7						
Elbow	APB	12.4	5.6	205		7.08		28.9	
Right ulnar nerve									
Wrist	ADM	4.01	9.8						
B. elbow	ADM	7.60	8.2	138		3.59		38.4	
A. elbow	ADM	10.68	8.1	102		3.07		33.2	
Axilla	ADM	13.80	7.2	125		3.12		40.0	
Right peroneal nerve									
Ankle	EDB	8.13	0.1						
Fib head	EDB	21.35	0.1	288		13.23		21.8	
Left peroneal nerve									
Ankle	EDB	9.11	0.3						
Fib head	EDB	19.17	0.3	298		10.05		29.6	
Right tibial nerve									
Ankle	AH	7.6	2.1						
Pop fossa	AH	23.39	0.9	365		15.78		23.1	
Left tibial nerve									
Ankle	AH	7.03	0.8						
Pop fossa	AH	21.09	0.8	365		14.06		26.0	

**F-wave**									
**Results**	**Side**					**Results**			

	Left		Right						
Tibial nerve									
Min F Lat ms	84.5		76.8			Min F Lat ms			
Max F Lat ms	90.9		85.3			Max F Lat ms			
Mean flat ms	87.9		80.4			Mean Flat ms			
%F%	100		100			%F%			
Peroneal nerve									
Min F Lat ms	77.2		82.0			Min F Lat ms			
Max F Lat ms	94.2		86.6			Max F Lat ms			
Mean flat ms	88.7		84.6			Mean Flat ms			
%F%	60		100			%F%			
Median nerve									
Min F Lat ms			39.2			Min F Lat ms			
Max F Lat ms			49.6			Max F Lat ms			
Mean flat ms			41.6			Mean Flat ms			
%F%			90			%F%			
Ulnar nerve									
Min F Lat ms			33.1			Min F Lat ms			
Max F Lat ms			45.1			Max F Lat ms			
Mean flat ms			34.5			Mean Flat ms			
%F%			100			%F%			

**Spontaneous potential**									
**Muscle**	**Fibrillation potentials**	**Positive sharp waves**	**Fasciculations**	**Muscle rigidity**	**Myokymic discharges**	**Complex repetitive discharges**			

Right tibialis anterior muscle	+	+	−	−	−	−			
Left gastrocnemius	+	−	−	−	−	−			
Right abductor pollicis brevis muscle	+	−	−	−	−	+			

*Note:* SCV, sensory nerve conduction velocity.

Abbreviations: CMAP, compound muscle action potential; MUP, motor unit potential.

### 2.6. Diagnoses and Treatment Plan

The patient’s admission diagnoses were CMT disease and type 2 diabetes. The treatment plan involves a combination of pharmacological interventions and rehabilitation therapies. The patient was administered mecobalamin 0.5 mg orally three times daily, vitamin B1 20 mg orally three times daily, vitamin C 0.1 g orally three times daily, and rat nerve growth factor 30 μg intramuscularly for nerve nutrition. Acarbose 50 mg orally three times daily with meals was prescribed to help control blood glucose levels. A comprehensive rehabilitation program was implemented, including acupuncture, electroacupuncture, moxibustion, traditional Chinese medicine fumigation, infrared therapy, electronic biofeedback therapy, and a power bicycle (THERA‐VITAL, Shanghai Huanxi Co., Ltd). Endurance training and exercise therapy were performed to improve muscle strength. Hyperbaric oxygen therapy was performed to improve myelin growth. The goal of this treatment plan was to manage the symptoms of CMT disease and type 2 diabetes, improve muscle strength and mobility, and enhance the patient’s overall quality of life. Regular monitoring and adjustments to the treatment plan were to be made based on the patient’s response and progress. After 2 weeks of treatment, the patient was discharged from the hospital.

## 3. Discussion

### 3.1. CMT Disease

Mutations in the *MPZ* gene (MIM ∗159440) on chromosome 1q23, which encodes myelin protein zero (P0), are associated with a spectrum of sensorineural neuropathies, including CMT disease, dominant intermediate D (CMTDID, #607791), CMT1B (#118200), CMT2I (#607677), CMT2J (#607736), DSS (#145900), congenital hypomyelinating neuropathy (CHN, #605253), and Roussy–Levy syndrome (RLS, #180800). Myelin protein zero (P0) is the major structural protein of peripheral myelin, accounting for more than 50% of the total protein in the peripheral nerve sheath. Expression of the *MPZ* gene is restricted to Schwann cells. *MPZ* is not found in the central nervous system. *MPZ* is thought to link adjacent lamellae and thereby stabilize myelin assembly. The other three major components of myelin are myelin basic protein (MBP), myelin proteolipid protein (PLP1), and myelin‐associated glycoprotein (MAG) [7]. MBP is a major structural protein in the myelin sheath, contributing to its elasticity and stability. It helps maintain the integrity of the myelin structure and is involved in regulating myelin assembly and maintenance. PLP1 is a glycoprotein that makes up a significant portion of the myelin sheath’s lipid component. It is essential for the formation and maintenance of the myelin sheath and for the speed and efficiency of nerve impulse conduction. MAG is a glycoprotein that interacts with the axonal surface, influencing axon growth and survival. It is also involved in maintaining the myelin sheath and plays a role in immune regulation within the nervous system.

Most patients with CMT are classified as having CMT1 or CMT2 using a cut‐off value of 38 m/s for median NCV. However, in some CMT families, patients have motor median NCVs ranging from 25 to 45 m/s. These families have been reported as having CMT‐Int. A four‐generation Macedonian family carrying an *MPZ* gene mutation presented with variable severity and intermediate motor NCVs. Affected members showed a symmetric pattern of distal muscle atrophy, weakness, and sensory impairment in the lower limbs, and to a lesser extent in the upper limbs. Motor NCVs ranged from 24 to 41 m/s for the median nerve and from 33 to 48 m/s for the ulnar nerve. Nerve biopsy from two patients showed primarily axonal degeneration, along with areas of segmental demyelination and remyelination without onion bulb formation [8].

CMT1B usually begins in the first or second decade of life, starting in the feet and legs with a peroneal distribution, while upper limb involvement generally appears later. The disease is slowly progressive, with an insidious onset and variable severity. Peripheral neuropathy leads to distal limb muscle weakness and atrophy, resulting in a “steppage” gait and foot drop. Cold‐induced muscle cramps, distal sensory impairment, hyporeflexia, and areflexia are also common. Decreased motor NCV (< 38 m/s) is observed, along with hypertrophic nerve changes. Nerve biopsies reveal onion bulb formations, segmental damage and repair of myelin, and fewer myelinated nerve fibers. Some patients also have myelin outfoldings [9].

CMT2I is caused by *MPZ* gene mutations and follows an autosomal dominant pattern. It starts later in life, between ages 47 and 60. Patients experience distal muscle weakness and wasting, foot deformities, sensory loss, and reduced reflexes, but nerve conduction speeds are normal or only mildly slowed [10].

CMT2J, also due to *MPZ* mutations, appears in the 4th–5th decade of life and includes distinctive features, such as marked sensory problems, deafness, and pupil abnormalities. Nerve conduction speeds can range from below 38 m/s to normal. Sural nerve biopsies show clusters of remyelinating axons, indicating nerve damage and repair [10].

DSS neuropathy begins in infancy and can be inherited in either dominant or recessive patterns. Severe distal motor and sensory problems delay motor development and make walking difficult. Some infants have generalized low muscle tone. Additional features may include high‐arched feet, scoliosis, and unsteadiness due to sensory loss. Nerve conduction speeds are very slow, sometimes below 10 m/s, and biopsies show a severe loss of myelinated fibers. DSS can be caused by mutations in *MPZ*, *PMP22*, *PRX*, or *EGR2* [11].

CHN2 is an autosomal dominant disorder with early‐onset low muscle tone, severely delayed motor development, muscle weakness with absent reflexes, and very slow nerve conduction due to improper myelin formation. The degree of severity is highly variable; certain neonates exhibit joint contractures and respiratory insufficiency, whereas others are able to walk [12]. CHN and DSS look similar clinically, but the key difference is that CHN involves failure to form myelin properly from birth, whereas DSS involves ongoing breakdown and repair of myelin [13].

RLS is an autosomal dominant condition starting in infancy or childhood. Children walk late, are clumsy, and often fall. It features early, prominent unsteadiness followed later by mild motor problems. The disease progresses very slowly, and most patients remain able to walk throughout life [13, 14]. It resembles CMT1A in many ways, such as foot deformities, distal weakness and wasting, absent reflexes, and some sensory loss, but differs by including a static tremor of the arms and a staggering gait [15].

Considering the patient is a 43‐year‐old Chinese individual with late‐onset type 2 diabetes, slow disease progression, no accompanying manifestations, such as deafness or ataxia, no family history, and no nerve biopsy performed, we concluded that the patient likely belongs to the CMT‐Int category.

Mutations in the *MPZ* gene are associated with CMT, characterized by progressive slowing of nerve conduction and Schwann cell hypertrophy. The patient’s initial symptoms included a gradual onset of muscle wasting, weakness, and sensory impairment in the lower limbs. Genetic testing identified a heterozygous mutation in the *MPZ* gene, specifically *MPZ* (NM_000530.6): c.548G > A (p.Trp183∗). This alteration results in replacement of the 183rd amino acid (tryptophan) by a stop codon; this premature stop codon is predicted to escape nonsense‐mediated mRNA decay, resulting in a C‐terminal truncation that may exert a dominant‐negative effect. This particular pathogenic mutation has not been documented before; therefore, the variant was classified as likely pathogenic according to ACMG evidence criteria (PVS1 + PM2). However, a different variant affecting the same codon (NM_000530.8 c.549G > A, p.Trp183∗, VCV000917145.1) has been documented as likely pathogenic in the ClinVar database (submitted by the Molecular Genetics Laboratory, London Health Sciences Centre, in June 2020).

The concept of dosage sensitivity of the *MPZ* gene is crucial for understanding the phenotypic variability associated with mutations in this gene. The *MPZ* gene is dosage‐sensitive, meaning that the amount of functional *MPZ* protein is critical for normal myelination and nerve function. In the context of *MPZ* mutations, most truncating mutations, which result in the production of a shortened, nonfunctional protein, are associated with severe forms of peripheral neuropathy. These mutations often occur in the terminal or penultimate exons of the *MPZ* gene and are not subject to nonsense‐mediated decay (NMD), a cellular pathway that degrades mRNA carrying premature stop codons. As a result, they are stably translated into mutant proteins with potential dominant‐negative activity, meaning they can interfere with the function of the normal *MPZ* protein [16]. The c.548G > A (p.Trp183Ter) variant identified in this case is situated at chr1:161276154 (GRCh37/hg19), approximately 100 bp from the exon 4 (chr1: 161276119–161276254) splice junction. Of note, *MPZ* consists of six coding exons, with exon 4 encoding the transmembrane domain. According to the classical “50–55 nucleotide rule,” a premature termination codon (PTC) situated ≤ 50–55 nt from the last exon–exon junction typically escapes NMD. However, a seminal study by Inoue et al. demonstrated that *MPZ* does not strictly obey this rule. A PTC located 2 bp from the end of exon 4 (Q215X) escaped NMD, whereas another PTC located 28 bp further upstream (G206X) triggered NMD [17]. Regardless of whether the classical “50–55 nucleotide rule” or the exception proposed by Inoue et al. (2004) is applied, our variant is located at a comparable or more upstream position and is therefore predicted to trigger NMD [17].

High‐resolution MRI has demonstrated lumbar and sacral nerve changes in CMT patients. In CMT1A, nerve thickening (moderate or greater in 65.2% of patients) decreases from proximal to distal segments, reflecting extensive demyelination and myelin sheath proliferation [18, 19]. In a CMT1B patient with an *MPZ* mutation, MR neurography revealed hypertrophy of the brachial and lumbosacral plexuses, with cauda equina and nerve root enhancement [20]. A study analyzing the imaging manifestations of CMT1A found that CMT1A patients’ nerves exhibited varying degrees of thickening, with a significant proportion (65.2%) showing moderate or greater thickening [20]. Based on the above findings, we speculate that similar changes may be present in the peripheral nerves of our patient.

### 3.2. CMT and Type 2 Diabetics

The comorbidity of CMT disease and type 2 diabetes exacerbates peripheral nerve injury [21]. Both diseases are common causes of peripheral neuropathy, and when they coexist, they produce an additive effect. The mutual aggravation between CMT and diabetes may involve the three mechanisms. Firstly, CMT itself causes hereditary peripheral neuropathy, while the metabolic abnormalities induced by diabetes (hyperglycemia, oxidative stress, microvascular disease) inflict additional damage on already vulnerable nerves. Secondly, in CMT patients, progressive muscle atrophy reduces skeletal muscle mass, which may exacerbate insulin resistance and impair blood glucose control. Thirdly, CMT’s inherent defects in myelination or axonal regeneration, combined with diabetic neuropathy, lead to a further decline in repair capacity.

In a study by Chan et al., the researchers examined two siblings, a brother and a sister, who both had CMT1A, type 2 DM, and diaphragmatic dysfunction [22]. Another study suggests a potential genetic association between CMT1A and type 2 DM within a large family of 69 members. In this family study, 28 members were found to have CMT1A, and molecular genetic studies were performed on 22 of them, revealing the chromosomal duplication in all cases. Additionally, six members with CMT1A also had type 2 DM, as defined by the American Diabetes Association diagnostic criteria. While the occurrence of both CMT1A and type 2 DM in this family could be coincidental, the family’s high prevalence of CMT1A, along with the presence of type 2 DM in a significant proportion of those with CMT1A, suggests a possible genetic link between the two conditions. Currently, the expression level of the *MPZ* gene in the pancreas is extremely low, making it unlikely to directly affect beta‐cell function. A more plausible explanation is an indirect mechanism: Chronic neuropathy and neuropathic pain may affect the hypothalamic–pituitary–adrenal axis and sympathetic nervous system activity, thereby influencing insulin sensitivity [21]. Further mechanistic studies will be conducted in the future.

The patient had used a sodium‐glucose cotransporter 2 inhibitor (SGLT2i) before the worsening of CMT symptoms, accompanied by significant weight loss. At present, SGLT2 inhibitors are widely used in clinical practice due to their therapeutic effects in reducing blood sugar and providing cardiovascular benefits. However, SGLT2 inhibitors all have weight loss effects, mainly due to reduced adipose tissue, but may also be accompanied by a decrease in lean body mass, especially skeletal muscle and water content [23]. The Japanese expert committee recommended that elderly type 2 diabetes patients aged over 65 years with myopia should use SGLT2i cautiously. Therefore, we speculate that the patient experienced rapid weight loss and muscle mass decline after using SGLT2i, which may be another reason for the worsening of their CMT symptoms [24]. Therefore, we recommend that patients with CMT avoid using SGLT2i for glycemic control.

### 3.3. Management of CMT

For pain management in CMT disease, nonsteroidal anti‐inflammatory drugs, paracetamol, and selective serotonin reuptake inhibitors are commonly used [25]. Calcium channel *α*2‐δ ligands, including gabapentin and pregabalin, reduce neurotransmitter release by binding to calcium channels and are widely used in the treatment of peripheral pain syndromes [26]. Interval‐training cycling combined with creatine supplementation has been shown to significantly increase the composition of myosin heavy chain type IIa, thereby improving physiological, neuromuscular, and functional capacity while alleviating fatigue in CMT patients [27]. Exercise represents an effective intervention in early‐stage CMT. To delay disease progression, rehabilitation programs should incorporate muscle strengthening, stretching, aerobic conditioning, and proprioceptive training [28]. Strengthening exercises compensate for distal muscle weakness, whereas stretching preserves joint range of motion and prevents contractures [29]. Aerobic exercise maximizes muscle and cardiorespiratory function and helps prevent secondary muscle atrophy in neuromuscular disorders [30]. Proprioceptive training significantly enhances balance, gait, functional mobility, and musculoskeletal endurance [31]. Long‐term multimodal exercise programs are particularly effective at improving functional outcomes [32]. Hyperbaric oxygen therapy, administered as 100% oxygen at 1.3–2.5 ATA, has been proposed to modulate inflammatory processes and enhance tissue repair [33].

## 4. Conclusion and Limitations

After 2 weeks of treatment, the patient was discharged due to a lack of financial resources to continue care. Although her blood glucose was well controlled, she reported minimal symptomatic improvement, with progressive weakness in foot dorsiflexion and persistent fatigue. Given that CMT disease is a neurodegenerative disorder, long‐term and continuous management is essential. As no family members were affected by CMT and genetic testing was not performed on them, the de novo origin of the variant could not be confirmed and therefore remains unknown.

## Author Contributions

Lu Xia wrote the original draft and edited this manuscript; Xin Chen and Liyan Cai collected and curated clinical data; Kun Zhou conducted physical therapy and occupational therapy; Wei Huang performed EMG; Shinian Yang provided acupuncture therapy; and Yuanyuan Zhu contributed to patient recruitment and data acquisition.

## Funding

This research received no specific funding.

## Disclosure

All authors reviewed and approved the final manuscript.

## Ethics Statement

This study was approved by the Ethics Committee of Chengdu First People’s Hospital (No. 2024‐YNYJ‐36).

## Consent

The patient has provided written informed consent for the publication of her personal and clinical details in this study.

## Conflicts of Interest

The authors declare no conflicts of interest.

## Data Availability

The data that support the findings of this study are available upon request from the corresponding author. The data are not publicly available due to privacy or ethical restrictions.
